# Development and Validation of a Prediction Model for Acute Hypotensive Events in Intensive Care Unit Patients

**DOI:** 10.3390/jcm13102786

**Published:** 2024-05-09

**Authors:** Toshiyuki Nakanishi, Tatsuya Tsuji, Tetsuya Tamura, Koichi Fujiwara, Kazuya Sobue

**Affiliations:** 1 Department of Anesthesiology and Intensive Care Medicine, Nagoya City University Graduate School of Medical Sciences, 1 Kawasumi, Mizuho-cho, Mizuho-ku, Nagoya 467-8601, Japan; 2 Department of Materials Process Engineering, Nagoya University, Furo-cho, Chikusa-ku, Nagoya 464-8603, Japan

**Keywords:** acute hypotensive event, hypotension, intensive care unit, machine-learning, prediction model

## Abstract

**Background**: Persistent hypotension in the intensive care unit (ICU) is associated with increased mortality. Predicting acute hypotensive events can lead to timely intervention. We aimed to develop a prediction model of acute hypotensive events in patients admitted to the ICU. **Methods**: We included adult patients admitted to the Nagoya City University (NCU) Hospital ICU between January 2018 and December 2021 for model training and internal validation. The MIMIC-III database was used for external validation. A hypotensive event was defined as a mean arterial pressure < 60 mmHg for at least 5 min in 10 min. The input features were age, sex, and time-series data for vital signs. We compared the area under the receiver-operating characteristic curve (AUROC) of three machine-learning algorithms: logistic regression, the light gradient boosting machine (LightGBM), and long short-term memory (LSTM). **Results**: Acute hypotensive events were found in 1325/1777 (74.6%) and 2691/5266 (51.1%) of admissions in the NCU and MIMIC-III cohorts, respectively. In the internal validation, the LightGBM model had the highest AUROC (0.835), followed by the LSTM (AUROC 0.834) and logistic regression (AUROC 0.821) models. Applying only blood pressure-related features, the LSTM model achieved the highest AUROC (0.843) and consistently showed similar results in external and internal validation. **Conclusions**: The LSTM model using only blood pressure-related features had the highest AUROC with comparable performance in external validation.

## 1. Introduction

Patients in the intensive care unit (ICU) sometimes experience hypotension due to various causes, including infection, hemorrhaging, and compromised cardiac function. Persistent hypotension is associated with increased mortality and should be treated promptly [1,2,3]. Early identification of acute hypotensive events contributes to early assessment of the patient’s condition and timely intervention by administering fluid, vasopressors, and inotropic agents. Thus, predicting acute hypotensive events may improve the prognosis of patients in the ICU.

With the advancement of machine learning and deep learning in the field of healthcare, numerous prediction models have emerged to forecast patient deterioration or disease onset. Several prediction models have been developed to predict acute hypotensive events for ICU patients [4,5,6,7,8,9,10,11,12]. However, some challenges remain to be addressed from previous studies on developing prediction models for acute hypotensive events in ICU patients. First, most models were constructed using the freely available Medical Information Mart for Intensive Care (MIMIC) Clinical Database [4,5,6,7,8,9,11,12]. Moreover, only two studies have validated their models using a dataset from different countries or regions [8,10]. Thus, the generalizability of prediction models for acute hypotensive events in the ICU has not been well established.

In addition to developing and validating prediction models, models should be constructed for future use in clinical practice. Intensivists work in emergency rooms, operating rooms, and hospital wards, as well as the ICU. While the extensive utilization of smartphones enables remote monitoring of patient data, alarm fatigue caused by frequent alarms can pose a risk to patient safety [13,14]. Thus, a tool that predicts hypotensive events and remotely alerts them promptly without alarm fatigue should be developed.

In this study, we aimed to develop and internally validate a prediction model for acute hypotensive events in patients admitted to the ICU using a single-center, university hospital ICU cohort. We also aimed to externally validate the developed models using a geographically different cohort. Furthermore, we aimed to clarify valuable features to predict acute hypotensive events and simulate alarm frequencies for future implementation in a smartphone application.

## 2. Materials and Methods

### 2.1. Ethical Approval

The Nagoya City University Graduate School of Medical Sciences and the Nagoya City University Hospital Institutional Review Board approved our study protocol (60-21-0154, 22 March 2022). The manuscript was written in accordance with TRIPOD guidelines [15].

### 2.2. Study Design

This was a retrospective, observational study conducted to develop and validate the multivariate prediction models in the ICU cohort. The developed model was also validated with an extra dataset.

### 2.3. Data Source

The models were developed and validated using the single-center ICU cohort of the Nagoya City University (NCU) Hospital in Japan between 1 January 2018 and 31 December 2021. Data were extracted from electronic medical records for the ICU (Fortec ACSYS, Koninklijke Philips N.V., Amsterdam, The Netherlands). We used the MIMIC-III database for external validation. MIMIC-III is an extensive, freely available database comprising de-identified health-related data associated with >40,000 patients admitted to the Beth Israel Deaconess Medical Center’s (Boston, MA, USA) critical care units between 2001 and 2012 [16].

### 2.4. Inclusion and Exclusion Criteria

We included adult patients (≥18 years old) admitted to the NCU hospital’s ICU for at least 2 h with arterial pressure monitoring. Our ICU included medical and surgical patients. We used the same inclusion criteria to select patients for the external validation dataset and excluded those with unavailable vital signs or missing characteristics.

### 2.5. Outcome

Based on a discussion of four certified intensivists (T.N., T. Tsuji, T. Tamura, and K.S.) of the Japanese Society of Intensive Care Medicine, a 15 min gap window was considered sufficient to prevent hypotension after remote issuance of the alert. A hypotensive event was defined as a mean arterial pressure (MAP) < 60 mmHg for at least 5 min in a consecutive 10 min period [9]. Hypotensive events within 2 min were counted as the same [9].

### 2.6. Data Preprocessing

The following were used as predictors: age, sex, and time-series data on seven vital signs recorded every minute (systolic and diastolic blood pressure [SBP and DBP, respectively], MAP, pulse pressure [PP], heart rate [HR], peripheral oxygen saturation [SpO_2_], and respiratory rate [RR]). All blood pressure (BP) data were derived from arterial pressure monitoring. MAP was calculated as [SBP + (DBP × 2)]/3, and PP was calculated as SBP − DBP. We removed physiologically implausible values, such as SBP, DBP, or MAP < 10 or >400 mmHg, RR < 1 or >100/min, HR < 10 or >400 bpm, or SpO_2_ < 10% [9]. Moreover, we excluded samples with ≥10% missing data [10]. Missing time-series data of <10 min were imputed using the moving average of the previous 3 min’s data [9].

### 2.7. Input Features

Ten statistics, consisting of maximum, minimum, first, second, and third quartiles, standard deviation (SD), skewness, kurtosis, and slope and intercept from single regression analysis, were calculated for each of the seven vital sign’s time-series data. Input features used for the prediction model consisted of 72 variables, including age and sex. These features were selected because remote monitoring has already been established with a smartphone application.

A number of features should be removed to improve computational efficiency and generalizability. Thus, we also constructed a model using only BP-related variables (SBP, DBP, MAP, and PP) as input features. Furthermore, we constructed a model using a one-point MAP value 15 min before the hypotensive event to evaluate the efficacy of machine-learning models.

**Figure 1:**
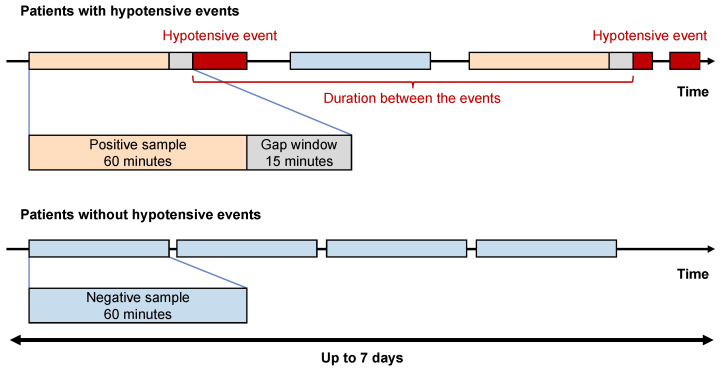
Definitions of positive and negative samples.

The patients were divided into two groups: those who developed hypotension at least once and those who did not. We defined a positive sample as 60 min of consecutive data up to 15 min before the hypotensive event (Figure 1). By contrast, we defined a negative sample as 60 min of consecutive data without hypotension after 15 min. Positive samples were extracted from hypotensive patients in as large a number as possible to avoid overlapping. Negative samples were randomly selected from patients who did and did not develop hypotension, in the same proportion as those who developed hypotension and in the same number as the positive sample. For patients admitted to the ICU for >7 days, data from the first 7 days were used to prevent a large number of samples from being taken from such patients.

### 2.8. Models

To develop a classification model for predicting acute hypotensive events, we compared three machine-learning algorithms: logistic regression (LR), the light gradient boosting machine (LightGBM), and long short-term memory (LSTM). In the LR and LightGBM models, 10 statistics of each vital sign were used as input features in addition to age and sex. In the LSTM model, 60 min time-series data on vital signs (maximum, minimum, mean, and SD), age, and sex were used as input features (Figure S1). We constructed models using all 72 and only 40 features related to BP for each machine-learning algorithm. In the one-point MAP model, the MAP threshold was determined to maximize the F1 score, the harmonic mean of precision and recall.

### 2.9. Training

The NCU hospital cohort was randomly split into 80% and 20% training and test datasets, respectively. In the LR model, grid search and fivefold cross-validation were used for hyperparameter tunings. In the LightGBM model, the Bayesian optimization-based hyperparameter search library (Optuna, Preferred Networks, Tokyo, Japan) [17] and fivefold cross-validation were used. The LSTM model was trained using the Adam optimizer to minimize the cross-entropy of the loss function. In all three models, hyperparameter tunings were performed to maximize the AUROC.

### 2.10. Validation

We validated the trained models using internal (hold-out test dataset of the NCU hospital) and external (MIMIC-III dataset) validation datasets. Subsequently, the models’ performances were evaluated using the area under the receiver-operating characteristic curve (AUROC), F1 score, accuracy, precision, recall, and specificity.

### 2.11. Alert Simulation

We evaluated the alert frequencies of the best performance model to evaluate clinical application. The decision to trigger the alert was simulated every minute using the holdout test dataset of the NCU hospital cohort. The alert decision was skipped if the patient was already developing hypotension, if there was ≥10% missing data in the observation window, or if the prediction score was <0.5. The frequencies of triggered alerts were calculated using lockout times of 30 and 60 min for alert suspension after the alert was triggered.

### 2.12. Statistical Analysis

We did not calculate the sample size a priori because of the large number of data used to develop and validate prediction models. Continuous and categorical variables were described as mean ± SD and median (interquartile range), and numbers (proportion), respectively. All computations were conducted by NTT DATA Japan Corporation (Tokyo, Japan) using Python (https://www.python.org/), scikit-learn (https://scikit-learn.org/stable/), and PyTorch (https://pytorch.org/); computer codes are not available due to confidentially.

## 3. Results

**Figure 2:**
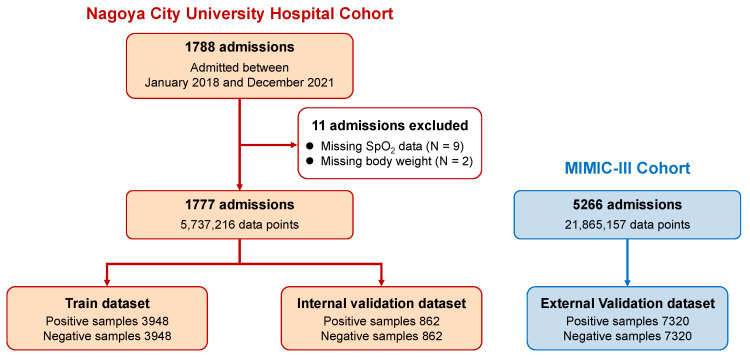
Flowchart of the study population for training and internal and external validation. MIMIC: Medical Information Mart for Intensive Care, SpO_2_: peripheral oxygen saturation.

We included 1777 admission data from 1600 unique patients aged 71 (59–78) years, of whom 35.5% were female, in the four-year NCU hospital cohort (Figure 2 and Table 1). For the external validation, 5266 admission data from 4758 unique patients aged 66 (55–76) years, including 41.4% female patients, were used from the MIMIC-III cohort. Acute hypotensive events were found in 1325 (74.6%) and 2691 (51.1%) cases in the NCU and MIMIC-III cohorts, respectively. Total hypotensive durations were 29 (17–61) and 23 (16–42) min, and durations between hypotensive events were 39 (10–158) and 53 (11–213) min in the NCU and MIMIC-III cohorts, respectively. The NCU hospital and MIMIC-III cohorts had 0.5–4.8% and 7.9–29% missing vital sign data, respectively (Tables S1 and S2). Compared with the NCU hospital cohort, the MIMIC-III cohort had slightly higher BP values and more missing and outlier data (Tables S1–S4).

We extracted 4310 positive and 4310 negative samples from the NCU cohort, with a 3:1 ratio of hypotensive and non-hypotensive cases, respectively, based on the proportion of hypotensive cases (74.6%). Of the 8620 samples, 6896 and 1724 were included in the training and validation datasets, respectively. From the MIMIC-III cohort, 7320 positive samples and the same number of negative samples (from hypotensive and non-hypotensive cases in a 55:45 ratio) were extracted as an external validation dataset. Tables S3 and S4 show the detailed vital sign values of training and validation samples.

**Figure 3:**
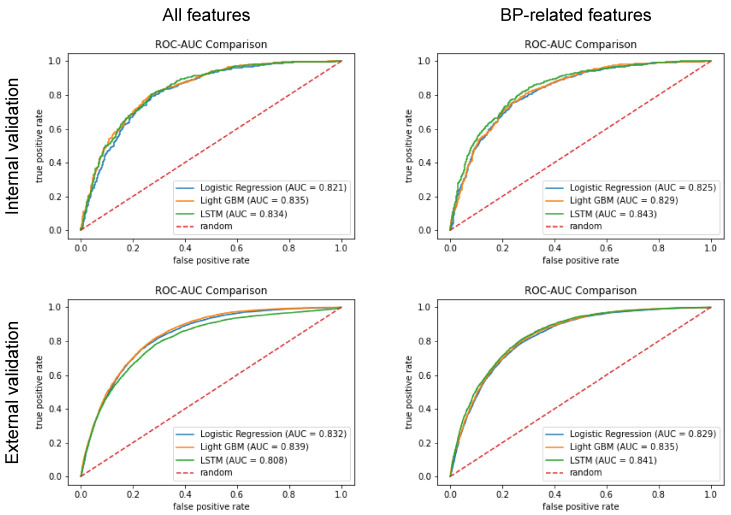
Comparison of models’ performances using all features or blood pressure-related features in internal and external validations. AUC: area under the curve, BP: blood pressure, LightGBM: light gradient boosting machine, LSTM: long short-term memory, ROC: receiver-operating characteristic.

In the internal validation using 72 features, the LightGBM model achieved the highest AUROC (0.835), followed by the LSTM (AUROC, 0.834) and LR (AUROC, 0.821) models (Figure 3 and Table 2). When applying only BP-related features, the LSTM model had the highest AUROC (0.843), which exceeded those of all models using 72 features.

External validation showed similar results to internal validation. Using all features, the LightGBM model had the highest AUROC (0.839). Using only BP-related features, the LSTM model had a higher AUROC (0.841) than all models using 72 features (Table 3). For internal and external validation, the machine-learning models had better performances than the model using 15 min earlier one-point MAP values of 77 mmHg (derived from the best F1 score).

The top four important features of the LightGBM model using 72 features were MAP-related features, including the minimum, first, and second interquartile MAP values, and MAP slope, in that order (Figure S2). The alert frequencies of the best performance model (LSTM using BP-related features) were 0.61 (0.34–0.85) and 0.39 (0.23–0.51) times/h with lockout times of 30 and 60 min, respectively.

## 4. Discussion

We developed and validated machine-learning models to predict acute hypotensive events in the ICU using the NCU hospital cohort of 1777 admissions and 5.73 million data points. Additionally, we performed external validation using 5266 admissions and 21.86 million data points from the MIMIC-III database. The LSTM model using only BP-related features achieved the best performance, with the highest AUROCs of 0.843 and 0.841 for internal and external validation, respectively. These results indicate that the developed models have good discrimination performance and generalizability.

Since the 2010s, several studies have developed prediction models for acute hypotensive events in patients admitted to the ICU. Early studies defined hypotension as MAP < 60 or 65 mmHg for >90% of 30 min based on the 10th Annual PhysioNet/Computers in Cardiology Challenge [4,5,7,11,12,18]. However, this definition should be revised and may miss clinically hypotensive events, which should be treated promptly, and the constructed model can lead to excessive false positives. Thus, the application of this definition to clinical practice may be challenging [10].

After a discussion with certified intensivists, we defined hypotension based on the study by Yoon et al. [9]. Our study was similar to that of Yoon et al. in terms of the balanced ratio of positive to negative samples and the gap window of 15 min. However, the performance of the random forest model developed by Yoon et al. (AUROC, 0.93 and area under the precision-recall curve, 0.77) was higher than ours, possibly because their goal was limited to predicting the first hypotensive event in the ICU. In contrast, our model addresses recurrent hemodynamic deterioration because we targeted the prediction of all hypotensive events during the first week of ICU admission.

We developed a model using a university hospital cohort in Japan and externally validated it using a large MIMIC-III cohort that was regionally and racially diverse. Only a few studies have reported on the external validation of prediction models for acute hypotensive events in ICUs. Cherifa et al. developed a model using the MIMIC-II cohort and externally validated it in their small cohort (N = 55) in France [8]. In the study by Cherifa et al., the input features used were severity scores, ventilatory status, and administration of vasopressors and sedatives to construct the super learner machine-learning model. Our model can be easily implemented clinically because it only uses vital signs, age, and sex. Because of the imbalanced ratio of positive to negative samples (80,667 vs. 17,472 and 426 vs. 12), AUROCs were high (0.929 and 0.884), and F1 scores were low (0.67 and 0.50) for internal and external validation, respectively [8]. Thus, their model cannot simply be compared to ours, which had a 1:1 ratio for positive and negative samples.

In our study, the LSTM, a deep-learning algorithm suitable for multivariate time-series data, achieved the best discrimination performance, consistent with that of Chan et al., which showed better performance of deep learning-based models than LR or support vector machine models [10]. The study by Chan et al. addressed hypotension individually and defined hypotension as the difference between the 60 and 5 min moving averages of ≥20% [10]. Because the relative rather than absolute threshold had no benefit for intraoperative hypotension, further validation is needed to determine the efficiency of predicting relative hypotension in the ICU for prognosis [19].

MAP statistics dominated the high feature important values of the LightGBM model. Indeed, the performance of the LSTM model using only BP-related features was superior to any model using all features. Our results indicate that time-series BP values are sufficient to predict acute hypotension events. Sufficient performance with BP-related features is useful for clinical implementation because it can reduce computational resources and prevent omissions due to missing measurements of other vital signs.

The alert frequencies of the best performance model were 0.61 and 0.39 times/h with lockout times of 30 and 60 min, respectively. Yoon et al. showed that the frequency of alarms with a 15 min lockout time was 0.79 times/h. Although we were unable to simulate a 15 min lockout period, similar results would have been obtained from our results with lockout times of 30 and 60 min. Future studies are needed to prospectively validate sensitivity and the false positive rate using machine-learning model-based alerts.

Our models were designed for the implementation phase of a smartphone application that remotely notifies doctors using an existing data collection infrastructure. Recently, the Acumen Hypotension Prediction Index (HPI, Edwards Lifesciences, Irvine, CA, USA), which can predict acute hypotensive events using the machine-learning model, became commercially available [20]. Although HPI systems have been validated with ICU settings [21,22], this system requires specific sensors for arterial waveform analysis. Our model can be easily implemented clinically because only time-series data for BP every minute are required, which is likely available in most ICU electronic records.

Our study’s strength was the large amount of high-quality institution data, with low missing data in the training dataset. Previous studies have developed models using the MIMIC database, which may have selection bias due to the high missing data rate. Second, we validated the models using the external MIMIC-III cohort and internal hold-out dataset. The performance of models with external validation was as high as that of models with internal validation was, despite geographical and racial differences. Thus, developed models had advantages in terms of generalizability and robustness.

Our study had limitations. First, we only compared gap windows for 15 min. Considering our hospital’s working conditions, we set the models’ task to predict hypotensive events 15 min in advance. Generally, the shorter the gap window, the higher the predictive performance expected, and vice versa [4,10,20]. The clinical application should determine the gap window. Nevertheless, 15 min is reasonable for assessing the patient’s condition and administering vasopressors or fluid boluses in ICUs. Second, we did not simulate an alert for a recall and false positive rate using the external dataset. Future studies should focus on alert performance. Third, we used minute-by-minute data as input features for the prediction models. Recently, time-series data with higher temporal granularity have become available with improved computer processing power [23]. Inputting these extensive data into deep-learning algorithms may improve prediction performance.

## 5. Conclusions

We developed machine-learning models to predict acute hypotensive events in the ICU with 1777 admission data from our university hospital. We also performed external validation with 5266 admission data from the MIMIC-III database. The LSTM model using only BP-related features best predicted hypotensive events with a good discrimination performance and comparable performance in external validation. These results indicate the high generalizability and robustness of the developed models, facilitating their implementation in clinical practice. Future research is necessary to validate the efficacy of the prediction model when implemented in a clinical setting. The outcomes should encompass the duration and severity of hypotension, as well as its impact on the patient’s prognosis.

## Figures and Tables

**Table 1:** Characteristics of the Nagoya City University Hospital and MIMIC-III cohorts.

	NCU Hospital	MIMIC-III	SMD
Number of stays (N)	1777	5266	
Number of patients (N)	1600	4758	
Age (years)	71 (59–78)	66 (55–76)	0.07
Sex (female, N/%)	630 (35.5%)	2183 (41.5%)	0.12
Number of hypotensive stays (N/%)	1325 (74.6%)	2691 (51.1%)	0.50
Number of hypotensive events (N)	11,610	19,311	
Number of hypotensive events per stay (times)	5 (2–11)	4 (2–9)	0.17
Hypotensive duration (min)	29 (17–61)	23 (16–42)	0.16
Duration between hypotensive events (min)	39 (10–158)	53 (11–213)	0.07

NCU: Nagoya City University, SMD: standard mean difference.

**Table 2:** Models’ performance of the internal validation using the Nagoya City University Hospital cohort.

Models	AUROC	F1 Score	Accuracy	Precision	Recall	Specificity
All features						
LR	0.821	0.768	0.744	0.701	0.849	0.638
LightGBM	0.835	0.773	0.765	0.745	0.804	0.725
LSTM	0.834	0.776	0.767	0.747	0.806	0.728
Only BP-related features						
LR	0.825	0.767	0.748	0.714	0.827	0.670
LightGBM	0.829	0.766	0.757	0.737	0.797	0.716
LSTM	0.843	0.776	0.768	0.751	0.802	0.735
One-point MAP value	-	0.681	0.701	0.658	0.706	0.557

AUROC: area under the receiver-operating characteristic curve, BP: blood pressure, LR: logistic regression, MAP: mean arterial pressure.

**Table 3:** Models’ performance of the external validation using the MIMIC-III cohort.

Models	AUROC	F1 Score	Accuracy	Precision	Recall	Specificity
All features						
LR	0.832	0.767	0.759	0.742	0.795	0.723
LightGBM	0.839	0.756	0.759	0.766	0.745	0.722
LSTM	0.808	0.725	0.736	0.756	0.696	0.776
Only BP-related features						
LR	0.829	0.761	0.757	0.750	0.773	0.742
LightGBM	0.835	0.752	0.756	0.765	0.741	0.772
LSTM	0.841	0.763	0.764	0.767	0.759	0.769
One-point MAP value	-	0.706	0.724	0.689	0.724	0.630

## Data Availability

Data presented in this study are available on reasonable request and approval of the Nagoya City University Graduate School of Medical Sciences and the Nagoya City University Hospital Institutional Review Board due to ethical reasons.

## Supplementary Materials

The following supporting information can be downloaded at: https://www.mdpi.com/article/10.3390/jcm13102786/s1, Figure S1. The architecture of the long short-term memory model, Figure S2. The top 20 feature importance values of the LightGBM model, Table S1. Minute-by-minute vital sign data of the Nagoya City University Hospital cohort, Table S2. Minute-by-minute vital sign data of the MIMIC-III cohort, Table S3. Positive and negative samples of the Nagoya City University Hospital cohort, Table S4. Positive and negative samples of the MIMIC-III cohort, Table S5. Alert frequencies of the simulation using the Nagoya City University Hospital cohort.
