# Supplementary material for: Development and Validation of a Prediction Model for Acute Hypotensive Events in Intensive Care Unit Patients
Source: J Clin Med. 2024 May 9;13(10):2786. doi: 10.3390/jcm13102786 (PMC11122431; doi:10.3390/jcm13102786)
Supplement: Supplementary file 1 [file jcm-13-02786-s001.zip › jcm-2963954-supplementary.pdf]

Table S1

Minute-by-minute vital sign data of the Nagoya City University Hospital cohort.

	N	Median (IQR)	Missing (N/%)	Outlier (N/%)
SBP	5,737,216	114 (98–131)	134,527 (2.3%)	291 (0.01%)
DBP	5,737,216	55 (48–64)	134,527 (2.3%)	780 (0.01%)
MAP	5,737,216	75 (66–85)	134,527 (2.3%)	484 (0.01%)
Pulse pressure	5,737,216	57 (45–71)	134,527 (2.3%)	-
Heart rate	5,737,216	83 (71–98)	26,644 (0.5%)	4,859 (0.08%)
SpO_2	5,737,216	98 (96–100)	276,151 (4.8%)	37 (0.0006%)
Respiratory rate	5,737,216	17 (14–22)	136,804 (2.4%)	50,822 (0.89%)

DBP: diastolic blood pressure, IQR: interquartile range, MAP: mean arterial pressure,

SBP: systolic blood pressure, SpO_2: peripheral oxygen saturation

Table S2

Minute-by-minute vital sign data of the MIMIC-III cohort.

	N	Median (IQR)	Missing (N/%)	Outlier (N/%)
SBP	21,865,157	116 (100–135)	6,306,607 (29%)	1,414,225 (6.5%)
DBP	21,865,157	57 (48–67)	6,306,607 (29%)	1,415,134 (6.5%)
MAP	21,865,157	77 (67–89)	6,306,607 (29%)	1,414,382 (6.5%)
Heart rate	21,865,157	86 (74–98)	1,737,047 (7.9%)	58,168 (0.3%)
SpO_2	21,865,157	99 (97–100)	1,734,634 (7.9%)	769,900 (3.5%)
Respiratory rate	21,865,157	20 (16–24)	1,932,285 (8.8%)	547,100 (2.5%)

DBP: diastolic blood pressure, IQR: interquartile range, MAP: mean arterial pressure, SBP: systolic blood pressure, SpO_2: peripheral oxygen saturation

Table S3

Positive and negative samples of the Nagoya City University Hospital cohort.

	N	Mean ± SD	Median (IQR)	Missing (N/%)
SBP				
Positive	257,109	109 ± 18	106 (97–118)	1,385 (0.54%)
Negative (hypotensive)	192,810	119 ± 20	117 (105–132)	1,025 (0.53%)
Negative (non-hypotensive)	64,225	128 ± 21	127 (113–141)	395 (0.62%)
DBP				
Positive	257,098	52 ± 10	51 (46–56)	1,393 (0.54%)
Negative (hypotensive)	192,806	58 ± 11	57 (51–64)	1,025 (0.53%)
Negative (non-hypotensive)	64,220	63 ± 12	62 (55–69)	395 (0.62%)
MAP				
Positive	257,105	71 ± 10	69 (65–75)	1,389 (0.54%)
Negative (hypotensive)	192,809	79 ± 12	77 (70–85)	1,022 (0.53%)
Negative (non-hypotensive)	64,223	85 ± 13	83 (76–92)	392 (0.61%)
Pulse pressure				
Positive	257,098	57 ± 17	55 (45–67)	1,396 (0.54%)
Negative (hypotensive)	192,806	61 ± 19	60 (48–73)	1,025 (0.53%)

Negative (non-hypotensive)	64,220	65 ± 19	64 (51–77)	395 (0.62%)
Heart rate				
Positive	257,900	82 ± 20	80 (67–93)	594 (0.23%)
Negative (hypotensive)	193,463	84 ± 20	81 (69–96)	368 (0.19%)
Negative (non-hypotensive)	64,457	76 ± 16	75 (64–86)	158 (0.25%)
SpO_2				
Positive	257,771	97 ± 4	98 (96–100)	723 (0.28%)
Negative (hypotensive)	193,353	97 ± 4	98 (96–100)	478 (0.25%)
Negative (non-hypotensive)	64,538	98 ± 2	98 (97–100)	77 (0.12%)
Respiratory rate				
Positive	257,294	17 ± 6	16 (13–21)	1200 (0.47%)
Negative (hypotensive)	193,059	18 ± 6	16 (13–21)	772 (0.40%)
Negative (non-hypotensive)	64,288	17 ± 5	15 (13–19)	327 (0.51%)

DBP: diastolic blood pressure, IQR: interquartile range, MAP: mean arterial pressure, SBP: systolic blood pressure, SD: standard deviation, SpO_2: peripheral oxygen saturation

Table S4

Positive and negative samples of the MIMIC-III cohort.

	N	Mean ± SD	Median (IQR)	Missing (N/%)
SBP				
Positive	433,829	111 ± 19	108 (99–121)	5,320 (1.23%)
Negative (hypotensive)	238,848	119 ± 20	116 (104–131)	2,699 (1.13%)
Negative (non-hypotensive)	195,613	133 ± 23	132 (117–148)	2,000 (1.02%)
DBP				
Positive	433,815	53 ± 11	51 (46–57)	5,334 (1.23%)
Negative (hypotensive)	238,846	58 ± 12	56 (50–64)	2,701 (1.13%)
Negative (non-hypotensive)	195,605	68 ± 14	66 (59–75)	2,008 (1.03%)
MAP				
Positive	433,827	72 ± 11	70 (65–76)	5,322 (1.23%)
Negative (hypotensive)	238,846	78 ± 12	76 (70–84)	2,701 (1.13%)
Negative (non-hypotensive)	195,604	90 ± 14	88 (80–98)	2,001 (1.02%)
Pulse pressure				
Positive	433,814	59 ± 18	58 (47–69)	5,335 (1.23%)
Negative (hypotensive)	238,846	61 ± 19	59 (48–73)	2,701 (1.13%)

Negative (non-hypotensive)	195,604	66 ± 20	65 (52–78)	2,009 (1.03%)
Heart rate				
Positive	438,791	86 ± 18	84 (73–96)	358 (0.08%)
Negative (hypotensive)	241,396	85 ± 17	84 (73–96)	151 (0.06%)
Negative (non-hypotensive)	197,478	85 ± 16	84 (73–96)	135 (0.07%)
SpO_2				
Positive	437,858	97 ± 4	98 (96–100)	1,291 (0.29%)
Negative (hypotensive)	240,924	97 ± 3	98 (96–100)	623 (0.26%)
Negative (non-hypotensive)	197,163	97 ± 4	98 (96–99)	450 (0.23%)
Respiratory rate				
Positive	437,821	19 ± 6	19 (15–23)	1,328 (0.30%)
Negative (hypotensive)	240,987	20 ± 6	19 (16–23)	560 (0.23%)
Negative (non-hypotensive)	197,200	19 ± 5	18 (15–22)	413 (0.21%)

DBP: diastolic blood pressure, IQR: interquartile range, MAP: mean arterial pressure, RR: respiratory rate, SBP: systolic blood pressure, SD: standard deviation, SpO_2: peripheral oxygen saturation

Table S5

Alert frequencies of the simulation using the Nagoya City University Hospital cohort

	Thirty minutes washout		Sixty minutes washout	
	Mean ± SD	Median (IQR)	Mean ± SD	Median (IQR)
One week from the admission				
All patients	0.61 ± 0.34	0.61 (0.34–0.85)	0.38 ± 0.19	0.39 (0.23–0.51)
Hypotensive patients	0.66 ± 0.32	0.65 (0.41–0.87)	0.40 ± 0.17	0.41 (0.27–0.52)
Non-hypotensive patients	0.47 ± 0.37	0.36 (0.17–0.71)	0.30 ± 0.20	0.26 (0.13–0.44)
All admission data				
All patients	0.61 ± 0.33	0.61 (0.35–0.84)	0.38 ± 0.18	0.39 (0.23–0.51)
Hypotensive patients	0.65 ± 0.31	0.65 (0.41–0.86)	0.40 ± 0.17	0.40 (0.27–0.52)
Non-hypotensive patients	0.47 ± 0.37	0.36 (0.17–0.71)	0.30 ± 0.20	0.25 (0.13–0.44)

All data were shown as time/hour. IQR: interquartile range, SD: standard deviation

Figure S1

The architecture of the long short-term memory model

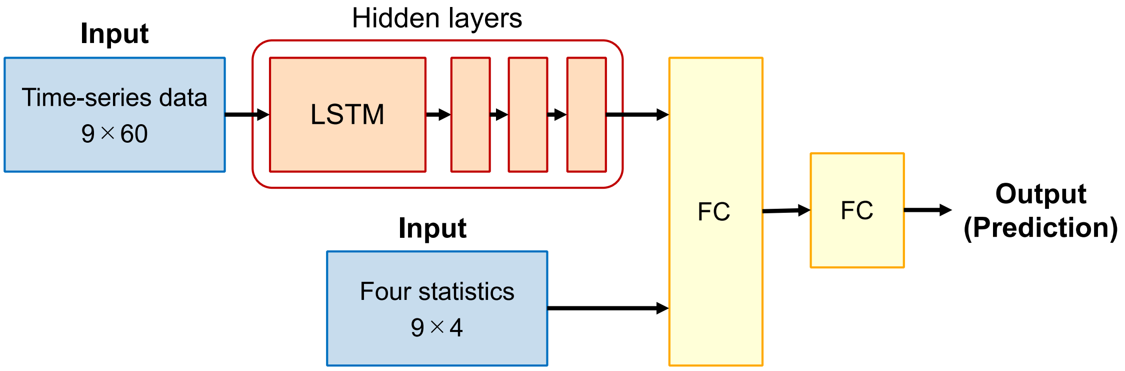

Input time-series data comprised 60 min of seven vital signs (systolic and diastolic blood pressure, mean arterial pressure, pulse pressure, heart rate, peripheral oxygen saturation, and respiratory rate), as well as age and sex. We also used maximum, minimum, quartiles, and standard deviation as input features. Parts in this diagram were simplified and omitted due to confidentially.

FC: fully connected, LSTM: Long Short-Term Memory

Figure S2

The top 20 feature importance values of the LightGBM model

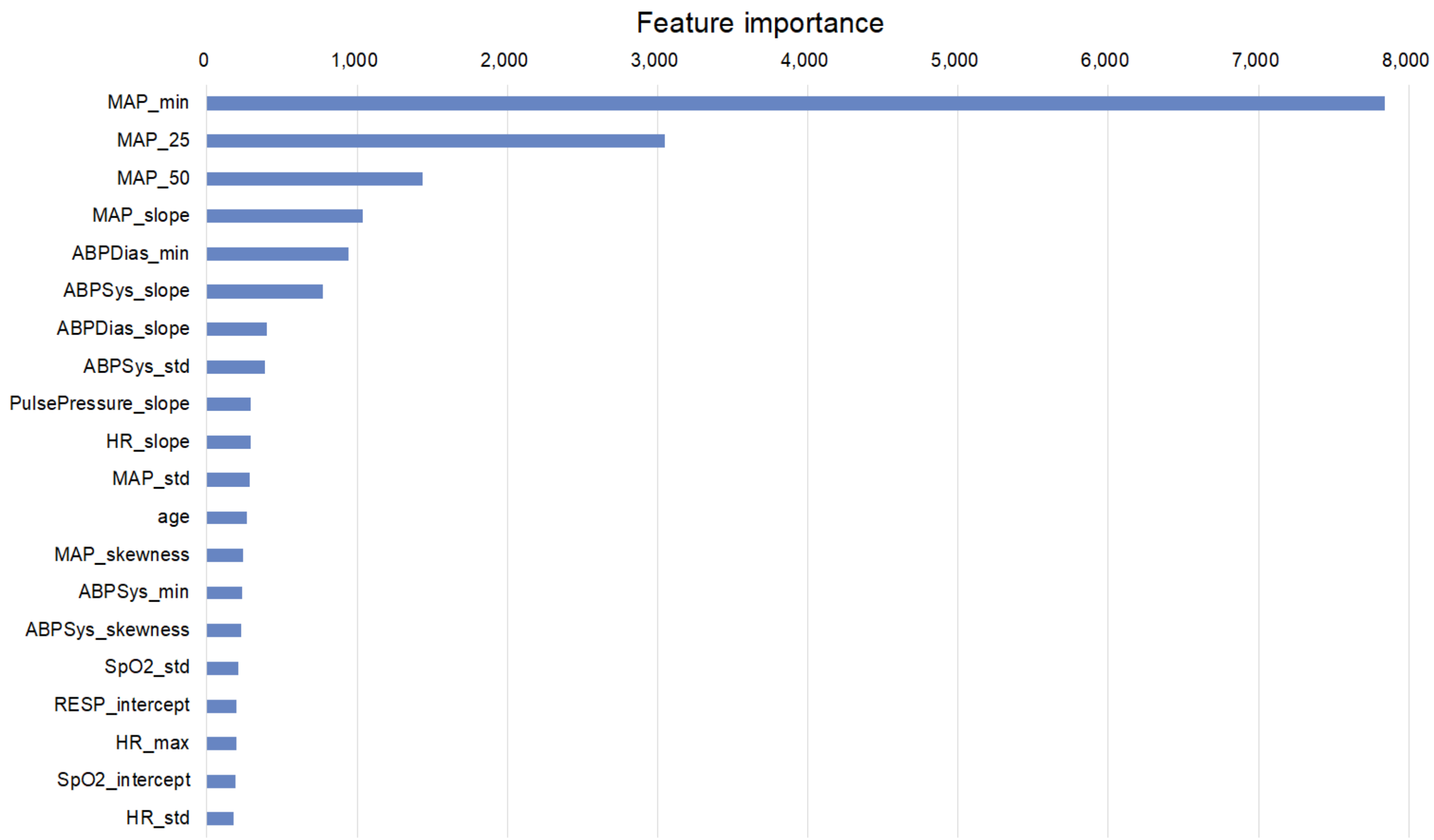

ABPDias: diastolic blood pressure, ABPSys: systolic blood pressure, HR: heart rate, MAP: mean arterial pressure, RESP: respiratory rate, SpO2: peripheral oxygen saturation

25: first quartile, 50: second quartile, intercept: intercept of the single regression analysis, max: maximum, min: minimum, slope: slope of the single regression analysis, std: standard deviation
